# Impact of Center-of-Mass Acceleration on the Performance of Ultramarathon Runners

**DOI:** 10.2478/hukin-2014-0109

**Published:** 2014-12-30

**Authors:** Shun-Ping Lin, Wen-Hsu Sung, Fon-Chu Kuo, Terry B.J. Kuo, Jin-Jong Chen

**Affiliations:** 1 Center for General Education, China University of Science and Technology, Taipei, Taiwan.; 2 Department of Physical Therapy and Assistive Technology, National Yang-Ming University, Taipei, Taiwan.; 3 Institute of Brain Science, National Yang-Ming University, Taipei, Taiwan.; 4 Department of Computer Science & Information Management, Soochow University, Taipei, Taiwan.

**Keywords:** accelerometer, race distance, ultra-marathon

## Abstract

Ultramarathon races are rapidly gaining popularity in several countries, raising interest for the improvement of training programs. The aim of this study was to use a triaxial accelerometer to compare the three-dimensional center-of-mass accelerations of two groups of ultramarathon runners with distinct performances during different running speeds and distances. Ten runners who participated in the 12-h Taipei International Ultramarathon Race underwent laboratory treadmill testing one month later. They were divided into an elite group (EG; n = 5) and a sub-elite group (SG; n = 5). The triaxial center-of-mass acceleration recorded during a level-surface progressive intensity running protocol (3, 6, 8, 9, 10, and 12 km/h; 5 min each) was used for correlation analyses with running distance during the ultramarathon. The EG showed negative correlations between mediolateral (ML) acceleration (r = −0.83 to −0.93, p < 0.05), and between anterior–posterior (AP) acceleration and running distance (r = −0.8953 to −0.9653, p < 0.05), but not for vertical control of the center of mass. This study suggests that runners reduce stride length to minimize mediolateral sway and the effects of braking on the trunk; moreover, cadence must be increased to reduce braking effects and enhance impetus. Consequently, the competition level of ultramarathons can be elevated.

## Introduction

Since a Greek runner Yiannis Kouros established the marathon record of 162.543 km over 12 h in New York, no one has surpassed this record for the past 30 years. This record is also a goal that numerous ultramarathoners have trained vigorously to pursue. Multiple factors affect the running economy of an ultramarathon, such as resistance of air (Costill and Fox, 1969), maximal aerobic power (Millet et al., 2011), muscle fiber distribution (Bosco et al., 1987; Millet et al., 2011), and the mechanical work and energy expenditure in kinematic measurement (Anderson, 1996; Cavanagh, 1990; Hausswirth et al., 1997; Kyrolainen et al., 2001; Loftin et al., 2007; Martin and Morgan, 1992; Mero et al., 1992; Novacheck, 1998; Williams and Cavanagh, 1986). However, Ronioyannis et al. (1989) emphasized the importance of energy balance of a runner during an extremely successful ultramarathon race as well as the methods through which their energy and nutrient requirements are fulfilled.

Previous research has suggested that ground reaction force (GFR), regardless of its magnitude or duration, is the main factor on which running speed depends (Roy, 1981; Hamill et al., 1983). To understand body displacement during physical activity, Belli et al. (1992) designed a “kinematic arm” consisting of four light rigid bars linked by three joints to conveniently observe and record the three-dimensional changes to the human body’s center-of-mass. In recent years, clinical physicians and researchers have utilized the widely popular accelerometer technology to quantify movement patterns; for example, in the neurological assessment of Parkinson’s patients (Fazio et al., 2012). Triaxial accelerometers provide detailed kinematic data on the human body (Wixted et al., 2010). They can measure the time course and mechanical characteristics of GFR in the stance phase, temporal and spatial variables of gait (Zijlstra and Hof, 2003; Moe-Nilssen and Helbostad, 2004). They are sensitive enough to detect rapid movements even if displacement is extremely small, such as involuntary tremor (Vaillancourt and Newell, 2000). The advantages of accelerometers over conventional gait analysis tools include low cost, small size, non-restriction of tests to the laboratory environment, and non-restriction of subject movement.

Studies utilizing triaxial accelerometers are now widespread and mature, and effectiveness of the technology is not limited to gait-related research. These instruments can measure the center-of-mass acceleration in three dimensions (Godfrey et al., 2008; Fazio et al., 2012). When used during an ultramarathon, they provide a simple method for the collection of gait quantification data during the running process with the aim of performing running-technique diagnosis and monitoring the effectiveness of running tactics. The aim of the present study was to use a triaxial accelerometer to compare the three-dimensional center-of-mass accelerations of two groups of ultramarathon runners with distinct performances during different running speeds and distances. These data provide invaluable information on the contribution of the center-of-mass acceleration to the performance of ultramarathon runners.

## Material and Methods

### Participants and experimental design

A total of 10 male members of the 12-h Taipei International Ultramarathon Race team volunteered to participate in the research. The subjects were characterized as follows: age, 50.30 ± 9.40 years; body height, 166.1 0 ± 6.04 cm; body mass, 59.5 ± 5.19 kg; BMI, 21.60 ± 1.35 kg/m^2^; body fat %, 14.10 ± 3.43%; maximum oxygen uptake, 52.90 ± 7.70 ml/kg/min. They were divided into an elite group (EG; n = 5) and a sub-elite group (SG; n = 5) based on their ability to run a distance greater or less than 96.2 km, respectively. There was no significant difference (p > 0.05) between participants in either group with respect to the BMI, body fat %, or VO_2 max_. The participants had no history of neurological disease and no injuries. Informed consent was obtained after the nature of the study’s procedures had been fully explained and understood. The study was approved by the Ethics Committee of the Taipei Veterans General Hospital.

### Measurements of running speed during the ultramarathon

The performance of the participants during the Taipei International Ultramarathon Race was evaluated in terms of running velocity using the manual calculation by Staff. After removing values associated with rest activities (sleep, toilet, food, and drink), running speed was calculated every hour to generate a mean hourly value for the entire race. The performance of participants in the EG and SG group was 98.34– 118.8 km (average speed of 8.2–9.9 km/h) and 74.8–94.29 km (average speed of 6.23–7.97 km/h; Table 1).

### Measurements of axial acceleration

The triaxial accelerometer (Analog Devices, Inc.; Massachusetts, USA; ADXL330, ±3 g) used in this study had dimensions of 52 × 32 × 6 mm, and total weight of 10 g, including the battery, and a continuous monitoring time of more than 60 h (Kuo and Yang, 2009; Kuo et al., 2011). Its three axes measured the linear acceleration of the human body’s center of mass and consisted of the VT axis (vertical or Z-axis) that corresponded to the vertical sway direction of the human body, the AP axis (anterior–posterior or Y-axis) that corresponded to the anterior– posterior sway direction of the human body, and the ML axis (mediolateral or X-axis) that corresponded to the lateral sway of the human body. Triaxial accelerometers have been shown to capture walking and running patterns accurately (Le Bris et al., 2006; Rowlands et al., 2007).

**Figure. 1:**
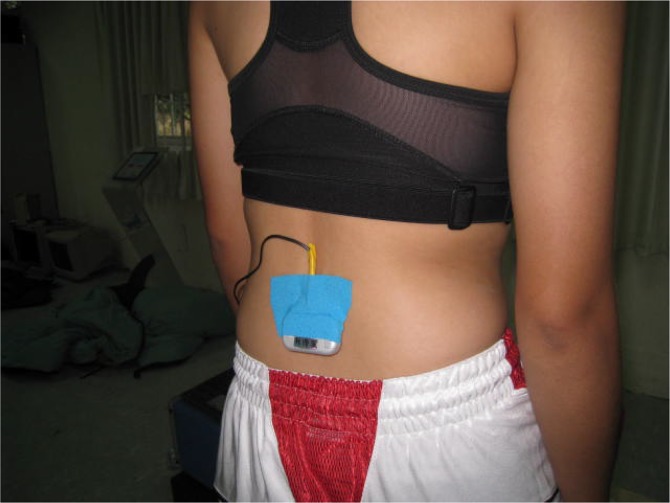
The accelerometer was worn at the height of the human body’s center of mass with kinesio tape.

The accelerometer was worn at the height of the human body’s center of mass, determined for each individual based on human anatomy and anthropometry (Godfrey et al., 2008; Kizilova et al., 2009). In this study, we attached the triaxial accelerometer at the intersection where the anterior superior iliac spine line connected with the vertebrae by using a highly cohesive, comfortable, and air-permeable Kinesio tape (Kinesio Holding Co., Albuquerque, USA; Figure 1). Before testing, the participants were instructed to stand quietly for 1 min to initialize the accelerometer. The center-of-mass acceleration of each subject within each stage was calculated based on the mean acceleration recorded on all three axes over the 1 min period. The accelerometer had a sampling rate of 500 Hz and measurement data could be stored on an internal 2-GB micro SD memory card.

### Laboratory protocols and data processing

Each subject underwent two movement laboratory test sessions one month after participating in the Taipei International Ultramarathon. The first consisted of baseline measurements of the BMI and % body fat as well as of maximum oxygen uptake (VO_2 max_) using the Bruce exercise treadmill protocol (Bruce et al., 1949). In brief, the subject started running at 3 km/h on a 10% grade. At three minute intervals the incline of the treadmill increased by 2%, and the speed increased by 1–1.5 km/h. The test score was the time reached in the test, in minutes.

The second was a running movement test performed two weeks later. The participants performed level-surface progressive-intensity running on a treadmill (Parker, Alabama, USA) and underwent gas analysis (True One 2400, ParvoMedics Co., Ltd., Utah, USA). Running test speeds were set at 3, 6, 8, 9, and 12 km/h, with each stage lasting 5 min and with a 2 min rest interval between each stage. At each stage, a period of 3.5 min was allowed for physiological variables to stabilize, and then acceleration and oxygen uptake were synchronously recorded during 1 min. The gas analyzer, air pressure, temperature, and humidity were recalibrated before each subject was tested.

### Statistical analysis

All statistical analyses were performed using SAS for Windows version 8.0. All variables (ML, AP, VT; m/s^2^) were expressed as means and standard deviations. The following statistical tests were conducted: 1. Pearson’s product-moment correlations were used to test the correlation between mean accelerations at different running speeds on the treadmill and in the ultramarathon, in which participants had previously participated; 2. the Wilcoxon rank sum test was used to test for significant differences in acceleration between the EG and SG groups; the level of statistical significance was set at p < 0.05.

## Results

Performance of the runners participating in the study was initially compared based on data collected during the Taipei International Ultramarathon Race. The performance distance for the EG group was 98.34–118.8 km (mean velocity: 9.22 km/h, range: 8.34–10.11 km/h), compared to 74.8–94.29 km (mean velocity: 7.55 km/h, range: 6.79–8.22 km/h) for the SG group (p < 0.05). These data suggest that better performance of the EG group, in terms of running distance, was derived from their ability to maintain a higher average running speed.

**Figure 2:**
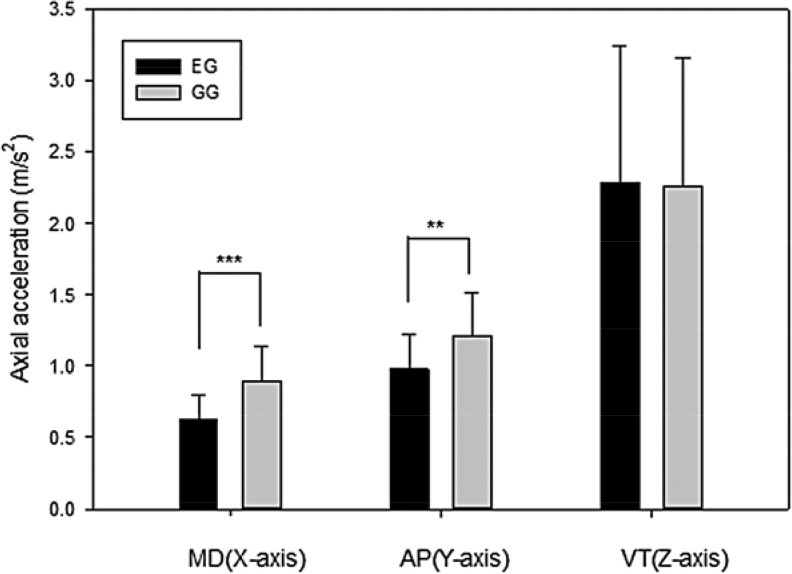
Axial acceleration differences between the Elite and Sub-elite groups. The critical role of the center-of-mass in the performance of ultramarathon runners was further demonstrated by comparing the axial acceleration rates between the EG and SG groups. Figure 1 shows that the EG group presented significantly lower center-of-mass acceleration rates in the ML (Z = −4.01, p < 0.0001) and AP axes (Z = −3.01, p < 0.01) than the SG group but not in the VT axis. These data are consistent with highly significant correlations detected between ML axial acceleration and running distance for the 10 runners (Table 2). These data suggest that runners selected for small lateral and anterior–posterior sway are more likely to perform better in long-distance races. * p < 0.05, ** p < 0.01, *** p < 0.0001

The triaxial accelerometer was used to conduct a three dimensional kinematic analysis of the center-of-mass acceleration during treadmill exercise. Measurements of axial acceleration were collected on all three axes over a range of running speeds (3–12 km/h) experienced by ultramarathon runners during a race. When the data from all 10 runners were combined, a negative correlation was detected between axial acceleration and running speed on the ML axis, which represents mediolateral sway, and running performance showed a significant correlation (r = −0.83 to −0.93, p < 0.05 to p < 0.01) at all speeds 3–12 km/h (ML3– ML12) for all participants (Table 2). These data suggest that mediolateral sway is the predominant factor affecting performance of the runners, reducing their ability to accelerate at high running speeds. The impact of the center-of-mass acceleration on the runners performance was first tested by comparing the EG group and SG group in terms of average acceleration on all three axes by the Wilcoxon rank sum test. Figure 2 shows that axial acceleration was significantly higher in the SG group on the ML axis (Z = −4.01, p < 0.0001) and the AP axis (Z = −3.01, p < 0.01). The components of the center-of-mass that affect the runners’ performance were identified by conducting correlation analyses between running speed and axial acceleration. The EG group exhibited significant correlations on the AP axis, which represents the anterior-posterior sway, with the highest coefficients at a running speed of 9–10 km/h (p < 0.0001). In contrast, the SG group presented only a weak correlation on the ML axis at a running speed of 6 km/h (p = 0.03) (Table 3), which represents mediolateral sway. These data suggest that anterior–posterior sway is the predominant factor affecting performance of elite runners, reducing their ability to accelerate at high running speeds.

The triaxial accelerator allowed us to determine the impact of running speed on the center of mass acceleration in all three axes with the treadmill protocol. When the data from all 10 subjects were compiled, no correlation was observed between race distance and the center-of-mass acceleration in the VT axis, regardless of the racing speed imposed by the treadmill (Table 2). With respect to the AP axis, the center-of-mass acceleration correlated with running speed only at low velocities (3–6 km/h). In contrast, the acceleration of the center-of-mass in the ML axis correlated negatively with running distance at all speeds (p < 0.003). These data suggest that mediolateral sway is the predominant factor affecting performance of runners, reducing their ability to accelerate at high running speed.

The correlation analysis was refined to determine whether the elite runners would adopt different strategies to control their center-of-mass better than the sub-elite runners. Table 3 shows that the center-of-mass acceleration was not a variable in the running distance achieved by the sub-elite runners. In contrast, the elite runners exhibited a significant correlation between running distance and the center-of-mass acceleration in the AP axis at nearly all speeds imposed by the treadmill. These data suggest that anterior–posterior sway is the predominant factor affecting performance of elite runners, reducing their ability to accelerate at high running speed.

The impact of running speed on the ability of EG and SG runners to stabilize their center-of-mass on each axis was investigated by the Wilcoxon rank sum test conducted between the mean axial accelerations of each group at speeds of 3–12 km/h. Table 4 shows that the EG group consistently maintained significantly lower axial accelerations on the ML axis at all running speeds (Z = −1.88 to −2.51, p < 0.05), with the highest stability at 8–10 km/h (Z = −2.51, p < 0.01). The AP axis also presented significantly higher axial accelerations for the EG group at low running speeds of 3,6 km/h (Z= −1.88 and −2.30; p < 0.05). Altogether, these data support a significant impact of the center-of-mass acceleration on ultramarathon runners’ performance. The EG group showed an overall higher ability to stabilize their core, especially along the ML and AP axes, especially when they reached an optimal running speed of 9–10 km/h. A gradual increase in the difference of the acceleration of the center-of-mass was observed as the running speed increased. These data suggest that runners selected for small lateral sway are more likely to exhibit superior performance in long-distance races.

The impact of running speed on the capacity of the athletes to control their center-of-mass was determined by comparing the mean axial acceleration rates of the EG and SG groups at 3–12 km/h. Table 4 shows that the EG group compared with the SG group presented significantly lower center-of-mass acceleration rates in the ML (3–12) and AP axis (3, 6), but not in the VT axis. There was a gradual increase in the difference of the acceleration of the center-of-mass as the running speed increased. These data suggest that runners selected for small lateral sway are more likely to perform better in long-distance races.

## Discussion

The Taipei International Ultramarathon Race at Soochow University is one of the highest-level competitions in the world. Since it takes place on a standard international 400 m track, runners can very easily control their running speed and compete to the best of their ability. This race was a unique opportunity to identify two distinct groups of ultramarathon runners in terms of running performance: the EG group and the SG group. The selected runners participated in laboratory tests evaluating the potential of triaxial accelerometers to identify the kinetic variables that determine their performance for the first time. The present study offers an in-depth analysis of the center-of-mass acceleration in all three axes, and their impact on running distances covered by ultramarathon runners.

The capacity of a triaxial accelerator to identify the variables determining performance of ultramarathon runners was first demonstrated by the impact of running speed on the center-of-mass acceleration along the ML axis. The 10 runners exhibited a negative correlation between mediolateral sway and a running distance of 3–12 km/h over a period of 5 min. Novacheck (1998) indicated that in walking or running, the hip was adducted while the limb was loaded in the stance phase and abducted during swing. Hip motion in this plane mirrors the movement of the pelvis, thereby minimizing shoulder and head movement. This is a very important mechanism for reducing intense lower extremity motion and allowing the head and trunk to maintain balance and equilibrium (Novacheck, 1998). Biomechanists have also verified that GRF is a major factor that affects running efficiency (Heise and Martin, 2001). As running speed increases, the difficulty of maintaining trunk and head balance also increases. This increase causes the mediolateral sway of the body to become more intense, resulting in rapid deterioration of running efficiency with increased running speed. This relationship suggests that maintaining pelvic stability (including maintaining balance and stability of the trunk and head) is an important technique for long-distance running. Our study indicates that at 6 and 8 km/h, there is strong correlation between ML axis acceleration (ML6, ML8) and running performance; this correlation is also strong at 9 km/h (Table 1). This result is in accordance with the range of 6–9 km/h for the running speeds of the participants in this study. Anderson (1996) indicated that elite runners were more capable at maintaining the trunk of the body at a vertical angle than sub-elite runners; hence, they were able to display more efficient kinematic performance in the latter stages of races. Some studies have indicated that elite runners not only had less vertical sway but also maintained upper-body symmetry and balance more easily (Novacheck, 1998). In accordance with the results of our study, elite runners are less inclined to manifest increased trunk sway with longer running times (fatigue may be a factor in this behavior), and this characteristic affects running performance.

The triaxial accelerator analysis also identified the AP axis as a significant determinant of performance of ultramarathon runners. Accordingly, our study further investigated the correlation between the center-of-mass acceleration and running performance at different test speeds among groups of different running abilities. There was a significant correlation between AP axis acceleration, which represents anterior–posterior sway, and running performance for EG group participants at speeds of 3–12 km/h (AP3, AP8–AP12); in particular, the correlation was even stronger at running speeds of 9 and 10 km/h (AP9, AP10) for most runners nearing the EG group level (Table 2). Research by Williams and Cavanagh (1987) indicated that elite long-distance runners did not only have smaller vertical GRF but also smaller anterior–posterior GRF. During gait analysis of running, Nummela et al. (2007) indicated that the ground contact phase was the only phase during which the runner could produce forces in various directions to affect running speed. The stance phase GRF is able to produce the characteristics of triaxial acceleration and reflect the functional and mechanical demands of the human body while running. As running speed increases, the vertical and horizontal components of GRF also increase (Mero et al., 1992; Weyand et al., 2000; Kyrolainen et al., 2001). Mero et al. (1992) additionally indicated that not only did the ground contact phase during running provide the runners propulsive force with every stride but the moment of ground contact also produced a braking force that affected running speed; they proposed that the horizontal distance between the first ground contact point and the center-of-mass of the body at touchdown should be very small to avoid loss of running speed and increased energy expenditure. Among the elite runners in our study, there was a strong correlation between AP axis acceleration, which represents anterior– posterior sway, and race performance. The lower a runner’s AP axis acceleration, the better was his running performance. This result is in line with the study by Mero et al. (1992).

Nummela et al. (2007) reported that horizontal GRF had linear correlations with running speed and maximal running speed. In our study, we further performed an independent-sample t-test on center-of-mass acceleration values on the ML and AP axes for the EG and SG groups at different running speeds. We discovered that for speeds of 3–12 km/h, there were significant differences in ML axis acceleration (ML3–ML12) between the EG and SG groups, particularly as speed increased, with the differences between the two groups becoming even more pronounced at 9 and 10 km/h (Table 3). We believe that most runners in the EG group had a running velocity of 9 or 10 km/h and sufficient ability to control body sway, and that it is for these reasons that ML axis acceleration of the EG group was markedly lower than that of the SG group. Our findings concur with those of Anderson (1996), who concluded that elite runners were more capable of maintaining the trunk of the body at a vertical angle than sub-elite runners, hence, they were able to display more efficient kinematic performance in the later stages of races. Our findings are in agreement with other studies stating that top-level runners not only have less vertical sway but also maintain upper-body symmetry and balance more easily (Novacheck, 1998). Other scientists have reported that the fore-after component associated with braking is characterized by a single or a double peak resulting from GRF at ground contact (Cavanagh and Lafortune, 1980; Hamill et al., 1983; Payne, 1983). Nummela et al. (2007) indicated that the horizontal component of ground reaction force of a distance runner’s center-of-mass was more important than its vertical component. Indeed in our study, VT acceleration did not attain significance in either correlation with running performance or differences between runners of different ability. These findings confirm that of the analyzed factors that affect running performance, ML and AP acceleration are more important than VT acceleration.

Based on the aforementioned results, we suggest that ultramarathon coaches and runners focus on reducing ML and AP-axis acceleration. Reducing ML-axis acceleration is related to reducing stride length and enhancing upper-body muscular strength and endurance. The reduction of AP-axis acceleration is related to reducing stride length and increasing cadence. Excess acceleration on the ML axis of a runner (mediolateral sway) could be caused by excess stride length or insufficient trunk strength of a runner. In addition to enhancing trunk strength and endurance, runners should examine whether their stride length is excessively long and appropriately reduce stride length to stabilize the trunk. An excessively high AP-axis acceleration (braking force) may be caused by excessive stride length, insufficient lower extremity press force, or both. Runners should reduce their stride length, increase their cadence, and strengthen their lower extremity press force. After a sufficient corpus of data is collected, real-time reminder devices can be designed to provide a comparison of results with standard norms as references for runners.

## Conclusion

ML and AP directions show strong correlations with running performance, indicating that elite ultramarathon runners show markedly lower directly measured center-of-mass acceleration than sub-elite ones. We suggest that runners reduce stride length to minimize mediolateral sway and the effects of braking on the trunk; moreover, cadence must be increased to reduce braking effects and enhance impetus. Consequently, the performance level of ultramarathon runners can be improved.

## Figures and Tables

**Table 1:** The anthropometric, physiological and velocity data for runners.

ID	Group	Age	Body height	Body mass	BMI	VO_2 max_	1st	2nd	3rd	4th	5th	6th	7th	8th	9th	10th	11th	12th	Performance
1	EG	50	173.0	68.3	22.8	60.2	9.92	9.09	9.50	9.09	9.50	7.85	7.85	7.85	7.85	8.26	7.02	8.26	102.06
2	EG	48	173.0	59.6	19.9	52.3	11.98	9.92	7.44	7.44	8.26	7.44	6.20	7.85	7.85	8.68	7.44	7.85	98.34
3	EG	60	161.0	57.5	22.2	47.1	9.92	9.92	9.50	9.09	9.09	8.26	9.50	8.68	8.68	6.61	8.68	8.90	106.83
4	EG	43	176.0	65.2	21.0	54.8	12.00	10.80	10.80	10.80	9.60	10.40	10.80	9.20	8.40	8.80	7.60	9.60	118.80
5	EG	35	161.0	57.3	22.1	72.0	12.19	10.61	10.80	11.20	9.20	10.40	10.00	9.20	8.00	8.00	7.20	9.20	116.00
6	SG	46	167.5	61.5	21.9	53.8	9.92	9.09	8.68	7.44	7.02	7.85	7.85	7.44	7.03	6.20	7.85	7.94	94.29
7	SG	41	160.5	48.1	18.7	61.5	9.50	8.26	8.68	7.85	8.68	7.02	6.61	7.02	4.96	2.89	7.85	9.11	88.44
8	SG	52	162.0	59.5	22.7	49.1	8.26	8.68	9.09	7.44	7.85	6.20	6.20	5.37	4.13	3.31	3.72	4.56	74.80
9	SG	59	167.0	59.8	21.4	38.4	8.68	7.44	7.02	7.02	7.02	6.20	6.61	7.02	5.37	4.96	6.20	6.43	79.98
10	SG	60	158.0	56.8	22.8	47.2	10.97	9.43	10.40	9.60	7.20	8.40	8.00	7.60	5.20	5.60	6.00	7.20	95.60

EG average		47.2	168.8	61.6	21.6	57.3	11.20	10.07	9.61	9.52	9.13	8.87	8879	8.56	8.16	8.07	7.59	8.76	108.41
SG average		51.6	163.0	57.1	21.5	50.0	9.47	8.58	8.77	7.87	7.55	7.13	7.05	6.89	5.34	4.59	6.32	7.05	86.62

**Table 2:** Impact of Running Speed on the Center-of-Mass Acceleration

	Running Speed (km/h)	Axial Acceleration (m/s^2^)	Correlation Factor (r)	p
ML (X-axis) (n = 10)	3	0.51 ± 0.10	−0.82	0.0031^**^
	6	0.60 ± 0.15	−0.93	<0.0001^***^
	8	0.73 ± 0.17	−0.92	<0.0001^***^
	9	0.82 ± 0.20	−0.87	0.0009^**^
	10	0.91 ± 0.22	−0.85	0.0018^**^
	12	1.02 ± 0.18	−0.92	0.0010^**^
AP (Y-axis) (n = 10)	3	0.88 ± 0.35	−0.68	0.0287^*^
	6	1.06 ± 0.31	−0.67	0.0314^*^
	8	1.06 ± 0.27	−0.21	0.5576
	9	1.11 ± 0.29	−0.19	0.5876
	10	1.18 ± 0.25	−0.09	0.7918
	12	1.27 ± 0.15	−0.52	0.1816
VT (Z-axis) (n = 10)	3	0.69 ± 0.11	−0.31	0.3819
	6	1.55 ± 0.23	−0.23	0.5114
	8	2.76 ± 0.28	−0.20	0.5648
	9	2.84 ± 0.2711	0.16	0.6576
	10	2.98 ± 0.18	0.22	0.5379
	12	2.94 ± 0.31	0.37	0.3632

* p < 0.05,

** p < 0.01,

*** p < 0.0001

**Table 3:** Impact of Running Speed on Center-of-Mass Acceleration for runners of different running abilities

	Running Speed (km/h)	Elite group (n = 5)			Sub-elite group (n = 5)		

		Axial Acceleration (m/s^2^)	Correlation Factor (r)	p	Axial Acceleration (m/s^2^)	Correlation Factor (r)	p
ML (X-axis)	3	0.44 ± 0.07	−0.39	0.51	0.58 ± 0.09	−0.86	0.05
	6	0.50 ± 0.08	−0.77	0.12	0.71 ± 0.13	−0.91	0.02^*^
	8	0.58± 0.05	−0.71	0.17	0.87 ± 0.11	−0.85	0.06
	9	0.64 ± 0.05	−0.81	0.09	1.00 ± 0.08	−0.42	0.47
	10	0.71 ± 0.06	−0.86	0.05	1.11 ± 0.08	−0.19	0.75
	12	0.89 ± 0.08	−0.68	0.19	1.22 ± 0.08	−0.67	0.20

AP (Y-axis)	3	0.65 ± 0.03	−0.92	0.02^*^	1.12 ± 0.36	−0.29	0.62
	6	0.82 ± 0.12	−0.80	0.09	1.29 ± 0.25	0.22	0.71
	8	0.98 ± 0.15	−0.93	0.01^*^	1.14 ± 0.36	0.49	0.39
	9	1.04 ± 0.21	−0.98	0.03^**^	1.17 ± 0.36	0.55	0.33
	10	1.14 ± 0.22	−0.96	0.01^**^	1.21 ± 0.31	0.69	0.18
	12	1.22 ± 0.16	−0.89	0.04^*^	1.35 ± 0.12	0.67	0.2081

VT (Z-axis)	3	0.66 ± 0.08	0.38	0.52	0.72 ± 0.14	−0.472	0.41
	6	1.44 ± 0.22	0.69	0.18	1.65 ± 0.22	−0.15	0.80
	8	2.71 ± 0.29	0.50	0.39	2.81 ± 0.30	−0.68	0.20
	9	2.85 ± 0.26	0.50	0.38	2.83 ± 0.31	−0.01	0.98
	10	2.99 ± 0.26	0.23	0.69	2.98 ± 0.09	0.63	0.24
	12	3.04 ± 0.34	0.20	0.74	2.77 ± 0.18	−0.56	0.33

* p < 0.05,

** p < 0.01,

*** p < 0.0001

**Table 4:** Axial acceleration differences between the Elite and Good groups at different treadmill running speeds

	Running Speed (km/h)	Axial Acceleration (m/s^2^)		Mann-Whitney U test (Z)	p
		Elite group (n = 5)	Sub-elite group (n = 5)		
ML (X-axis)	3	0.4434 ± 0.0749	0.5812 ± 0.0971	−1.88	0.0310^*^
	6	0.5033 ± 0.0860	0.7145 ± 0.1305	−2.09	0.0184^*^
	8	0.5899 ± 0.0571	0.8778 ± 0.1158	−2.51	0.0061^**^
	9	0.6404 ± 0.0598	1.0076 ± 0.0867	−2.51	0.0061^**^
	10	0.7177 ± 0.0611	1.1186 ± 0.0887	−2.51	0.0061^**^
	12	0.8990 ± 0.0836	1.2223 ± 0.0821	−2.09	0.0184^*^

AP (Y-axis)	3	0.6536 ± 0.0359	1.1238 ± 0.3699	−1.88	0.0301^*^
	6	0.8222 ± 0.1236	1.2988 ± 0.2578	−2.30	0.0108^*^
	8	0.9833 ± 0.1532	1.1469 ± 0.3620	−1.46	0.0718
	9	1.0456 ± 0.2128	1.1789 ± 0.3674	−1.04	0.1481
	10	1.1472 ± 0.2215	1.2142 ± 0.3161	−0.21	0.4173
	12	1.2251 ± 0.1667	1.3535 ± 0.1236	−1.19	0.1165

VT (Z-axis)	3	0.6603 ± 0.0833	0.7202 ± 0.1473	−0.42	0.3381
	6	1.4470 ± 0.2221	1.6576 ± 0.2283	−1.46	0.0718
	8	2.7168 ± 0.2925	2.8138 ± 0.3007	−0.63	0.2654
	9	2.8587 ± 0.2626	2.8391 ± 0.3100	0.0	0.5000
	10	2.9974 ± 0.2614	2.9802 ± 0.0911	0.21	0.4173
	12	3.0493 ± 0.3486	2.7772 ± 0.1857	1.19	0.1165

* p < 0.05,

** p < 0.01,

*** p < 0.0001
